# Echocardiography and Sports Cardiology: Expanding Horizons From Elite Athletes to Advanced Cardiovascular Disease

**DOI:** 10.31083/RCM53090

**Published:** 2026-07-23

**Authors:** Eduardo M. Vilela, Francisco Sampaio, José Ribeiro, Ricardo Fontes-Carvalho

**Affiliations:** ^1^Cardiology Department, Gaia Espinho Local Health Unit, 4434-502 Vila Nova de Gaia, Portugal; ^2^Faculty of Medicine, Porto University, 4200-319 Porto, Portugal; ^3^Unic@RISE, Faculty of Medicine, Porto University, 4200-319 Porto, Portugal

Sports cardiology has undergone major developments [1]. From the pre-participation assessment of athletes to risk stratification of individuals with cardiovascular disease (CVD), this field comprises a core component of cardiovascular medicine [2]. Inputs from areas such as genetics, non-invasive imaging, or invasive haemodynamic studies have provided insights into the association between cardiovascular physiology and exercise [2,3]. Given its availability, cost, portability, safety (namely absence of ionizing radiation), and versatility (allowing both rest and exercise workflows, and integration with other techniques), echocardiography stands as an important exam in sports cardiology, providing a comprehensive assessment of cardiovascular morphology and function (Fig. 1) [3,4,5].

**Fig. 1.**
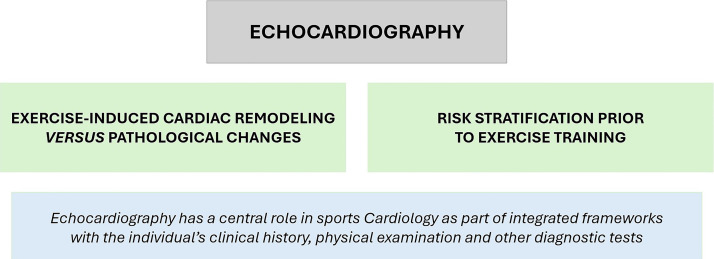
**General outline of some of the main applications of echocardiography in sports cardiology**. Echocardiography has a leading role in sports cardiology, as part of comprehensive workflows. These may include distinguishing between exercise-induced cardiac remodeling and pathology (particularly early-stage cardiomyopathies), and risk stratification of individuals with cardiovascular disease prior to exercise training. Some features of interest include chamber dimensions and wall thickness, aortic dimensions, valvular morphology and haemodynamic characteristics, or systolic and diastolic function (using a multiparametric approach across the examination). Both resting and stress (exercise) echocardiography can provide important data, the latter allowing an overview of the cardiovascular response to exercise. These should be analysed in view of the individual’s clinical history and physical examination, as well as other diagnostic tests (such as the electrocardiogram, but also other ancillary modalities according to the clinical context).

## 1. Exercise and the Cardiovascular System

Exercise may lead to several cardiovascular adaptations (classically described as the “athlete’s heart”) ranging from those on molecular and electrical levels, to functional and structural changes [1,3,6,7,8,9]. Different types of exercise impose a distinct burden on the cardiovascular system [3,10]. Although strict dichotomic classifications of endurance (isotonic) versus strength (isometric) training have major limitations, adaptations tend to be more prominent in sports with a pronounced endurance component, such as cycling or rowing [2,3,10]. These reflect the sustained increase in cardiac output (CO) with reduced or normal peripheral vascular resistance (PVR), whereas isometric training is characterized by slightly elevated or normal CO alongside significant (but typically transient) increases in PVR [5]. In this regard, harmonic dilatation of cardiac chambers, increases in left ventricle (LV) mass, and enhanced early diastolic filling (typically with a prominent E wave and an E/A ratio >2) have been reported [3,6,10,11]. These changes enable increases in stroke volume, addressing the need for higher CO coupled to improved performance [3,12,13]. Some adaptations, however, may lead to difficulties in phenotypic classification, namely with entities such as cardiomyopathies [14,15]. While not recommended for universal first-line screening of athletes, echocardiography is nonetheless pivotal when determining the degree of CVD [2,15,16].

## 2. Exercise-Induced Cardiac Remodeling and Differential Diagnosis

When differentiating physiological exercise-induced cardiac remodeling (EICR) from pathology, integrated frameworks are essential (**Supplementary Fig. 1**) [3,14,15,17]. Cardiovascular symptoms, such as chest pain or syncope with exertion, and physical examination abnormalities warrant further investigation [1,4]. Prior family history and electrocardiographic features also provide valuable information [1,17]. Furthermore, EICR may be influenced by age, sex, ethnicity, anthropometric data, training history, and the use of performance-enhancing or pharmacological agents [1,3,18,19].

Cardiac chamber dilatation is a hallmark of EICR [3,14]. Nonetheless, particularly in those with reduced left ventricular ejection fraction (LVEF), the presence of other entities such as dilated or arrhythmogenic cardiomyopathy may present challenges [1,14,20]. First, as stated above, clinical status and training history should be ascertained [1]. Second, unbalanced chamber dilatations (namely isolated right or LV dilatation) or findings such as complex ventricular ectopy or elevated natriuretic peptides, should prompt consideration of an underlying pathology [11,15,17,20]. Though applying specific cut-offs for LV dimensions is discouraged, evidence suggests that indexing dimensions to functional capacity may better reflect cardiac adaptations [3,12,15,21]. Interestingly, this indexing may also be relevant when assessing blood pressure, particularly in the context of hypertensive responses to exercise [22]. Finally, some highly conditioned athletes may exhibit a resting LVEF slightly below or close to the lower limit of normal. However, marked reductions in LVEF (particularly <45%) or impaired LV global longitudinal strain raise the possibility of disease [1,15,19,23]. Exercise stress echocardiography (ESE) can be useful in borderline cases, as an increase in LVEF (with cut-offs >11% and peak LVEF >63% having been described) supports physiological adaptations [1,4,20].

Slight increases in LV mass are also a feature of EICR [3,10,15]. These conditions optimize cardiac mechanics, thereby limiting excessive LV wall stress as this chamber dilates [10]. While acknowledging variations due to several factors (including sex and ethnicity, with higher values reported for male patients and those of African ancestry), marked LV hypertrophy (particularly LV wall thickness >15 mm) or asymmetric patterns should raise suspicion for hypertrophic cardiomyopathy (HCM) [1,3,6]. As previously noted, supranormal LV diastolic function is a feature seen in EICR [11]. Although E/A ratios and left atrial dimensions present pitfalls (with increases potentially linked to EICR), e’ and E/e’ may be useful when distinguishing physiological adaptations from pathology [11,14]. Despite their potential, current guidance does not include left atrial strain parameters in this context [11,15,23].

## 3. Risk Stratification—Focus on a Personalized Approach

An important application of echocardiography involves risk stratification prior to exercise training, where both resting and ESE can be of value [1,2,3,4,24,25,26,27]. While past paradigms were relatively restrictive, namely in settings such as HCM or congenital heart disease (CHD), current guidance supports personalized approaches on a background of shared decision-making [2,24]. Parameters such as LV systolic function are crucial when assessing patients with conditions ranging from chronic coronary syndromes to CHD [2]. This is also the case in valvular heart disease (VHD), where echocardiography provides data concerning morphology, functional status, and signs of extravalvular cardiac disease [2,24,28]. A thorough assessment is essential in arrhythmias to identify structural heart disease [2,24].

Regarding ESE, applications include analysing obstruction in HCM (with both diagnostic and prognostic value) or ischaemia in congenital abnormalities of the coronary arteries, myocardial bridging, or those with suspected chronic coronary syndromes [2,5,24,25,26]. As discussed in the current guidelines, ESE may also be valuable in VHD, namely in asymptomatic patients or in cases of mismatch between symptoms and resting findings [28]. By using exercise as a stressor (as opposed to drugs), ESE can be particularly useful in these settings to reproduce the physiological response to exertion, while data attests to its safety [2,5,19,27]. Beyond imaging, data concerning symptoms, functional capacity, blood pressure, and arrhythmias during ESE provide critical inputs for clinical decision-making [2,5,25,26].

## 4. Current Paradigms and Future Directions

Sports cardiology has evolved into a broad field, and several concepts derived from the cardiovascular response to exercise have extensive applications across the cardiovascular continuum [1,2,3,4,5,24,26,28]. As a highly adaptable test, echocardiography stands as a cornerstone in this field, assisting in harnessing the benefits of exercise while mitigating risks [2,3,4,5]. Whilst acknowledging limitations such as optimal pre-participation workflows or the long-term significance of findings such as LV dilatation, new developments continue to expand on previous results [16,17,29]. Notably, while addressing pitfalls such as validation, standardization, and population representation, artificial intelligence-assisted image interpretation could further streamline results, with deep learning techniques enhancing data integration [30]. As our understanding of the complex interaction between exercise and the cardiovascular system continues to advance, echocardiography is set to remain at the forefront of this challenging field.

## Abbreviations

CO, cardiac output; CVD, cardiovascular disease; CHD, congenital heart disease; EF, ejection fraction; EICR, exercise-induced cardiac remodeling; ESE, exercise stress echocardiography; HCM, hypertrophic cardiomyopathy; LV, left ventricle; LVEF, left ventricular ejection fraction; PVR, peripheral vascular resistance; VHD, valvular heart disease.
