# Supplementary material for: Echocardiography and Sports Cardiology: Expanding Horizons From Elite Athletes to Advanced Cardiovascular Disease
Source: Rev Cardiovasc Med. 2026 Jul 23;27(7):53090. doi: 10.31083/RCM53090 (PMC13419963; doi:10.31083/RCM53090)
Supplement: Supplementary file 1 [file 2153-8174-27-7-53090-s1.zip › Supplementary Material.docx]

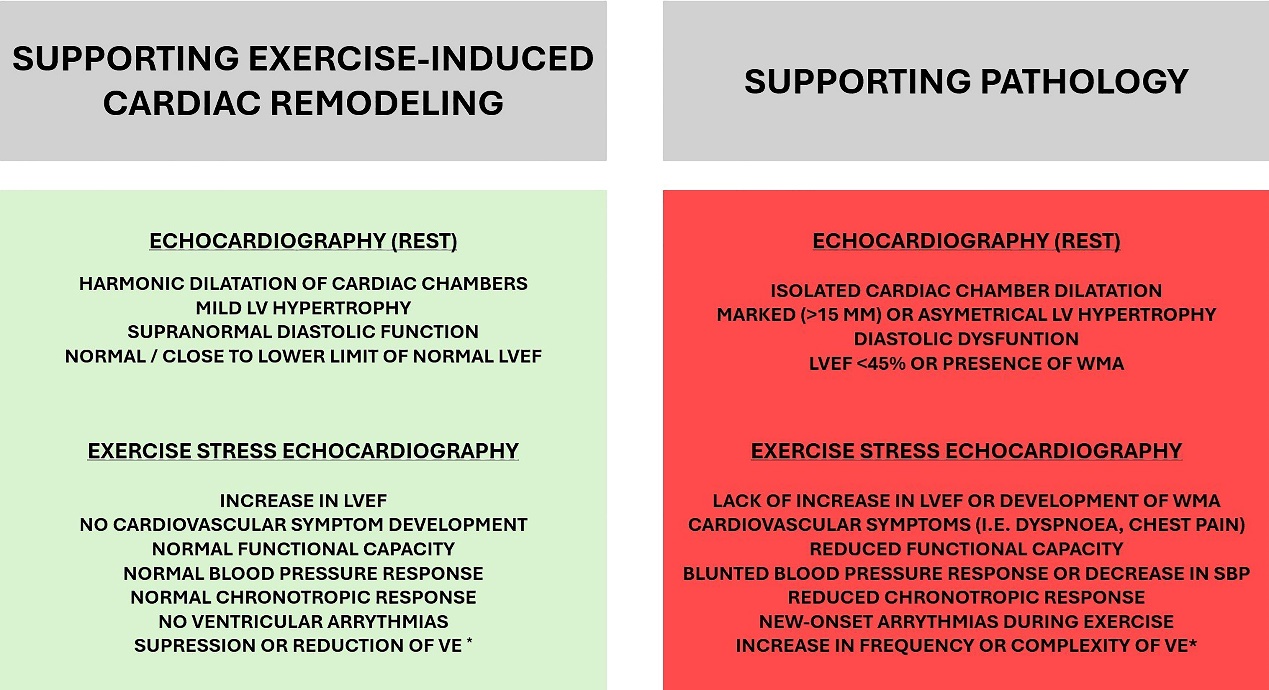


**Supplementary Fig. 1. Overview of echocardiographic features (both resting and during exercise) suggestive of exercise-induced cardiac remodeling, as opposed to underlying pathology.** Exercise training can lead to several cardiovascular adaptations, some of which may present varying degrees of overlap with underlying cardiovascular disease, such as cardiomyopathies. Both resting and exercise stress echocardiography may provide important data when assessing athletes, to allow accurate phenotyping and thus ensure appropriate management. Notably, beyond imaging data, exercise stress echocardiography also entails data on cardiovascular symptoms during exertion, functional capacity, blood pressure, and chronotropic response or the development of arrhythmias, all of which should be integrated to maximize the findings of this exam. Figure legend: LV, left ventricle; LVEF, left ventricular ejection fraction; SBP, systolic blood pressure; VE, ventricular ectopy; WMA, wall motion abnormalities; * , in those with baseline (resting) ventricular ectopy.
